# Identification and characterization of a fusarium head blight resistance gene *TaACT* in wheat QTL‐2DL

**DOI:** 10.1111/pbi.12641

**Published:** 2016-11-04

**Authors:** Udaykumar Kage, Shailesh Karre, Ajjamada C. Kushalappa, Curt McCartney

**Affiliations:** ^1^ Plant Science Department McGill University Sainte Anne De Bellevue QC Canada; ^2^ Agriculture and Agri‐Food Canada Morden Research and Development Centre Morden MB Canada

**Keywords:** Wheat, *Fusarium graminearum*, Metabolomics, Quantitative resistance, Near‐isogenic lines, Gene silencing

## Abstract

Fusarium head blight (FHB) resistance in wheat is considered to be polygenic in nature. Cell wall fortification is one of the best resistance mechanisms in wheat against *Fusarium graminearum* which causes FHB. Metabolomics approach in our study led to the identification of a wide array of resistance‐related (RR) metabolites, among which hydroxycinnamic acid amides (HCAAs), such as coumaroylagmatine and coumaroylputrescine, were the highest fold change RR metabolites in the rachis of a resistant near‐isogenic line (NIL‐R) upon *F. graminearum* infection. Placement of these metabolites in the secondary metabolic pathway led to the identification of a gene encoding agmatine coumaroyl transferase, herein referred to as *TaACT*, as a candidate gene. Based on wheat survey sequence, *TaACT* was located within a FHB quantitative trait loci on chromosome 2DL (FHB QTL‐2DL) between the flanking markers WMC245 and GWM608. Phylogenetic analysis suggested that *TaACT* shared closest phylogenetic relationship with an *ACT* ortholog in barley. Sequence analysis of *TaACT* in resistant and susceptible NILs, with contrasting levels of resistance to FHB, led to the identification of several single nucleotide polymorphisms (SNPs) and two inversions that may be important for gene function. Further, a role for *TaACT* in FHB resistance was functionally validated by virus‐induced gene silencing (VIGS) in wheat NIL‐R and based on complementation studies in Arabidopsis with *act* mutant background. The disease severity, fungal biomass and RR metabolite analysis confirmed *TaACT* as an important gene in wheat FHB QTL‐2DL, conferring resistance to *F. graminearum*.

## Introduction

Fusarium head blight (FHB) is a worldwide wheat disease caused by *Fusarium graminearum* that significantly affects yield and grain quality (Bai and Shaner, 1994, 2004; Dexter *et al*., 1996; Steiner *et al*., 2004). Many FHB QTL have been identified but the genes and their functions are largely unknown. Thus, there is an urgent need to identify the resistance genes underlying the QTL and to decipher the resistance mechanisms for their use in breeding programs. The FHB QTL on chromosome 2DL identified from Wuhan‐I × Nyubai (Somers *et al*., 2003) is one of the major QTL conferring rachis resistance (type II resistance) by limiting the spread of pathogen from the initial point of infection.

Despite significant efforts to identify and characterize the genes underlying FHB QTL using different tools, very few have led to new insights. Positional cloning of the QTL‐Fhb1 region of chromosome 3B disclosed the presence of seven novel genes in the 261‐kb region. Transgenic wheat lines were developed for four of these genes but none of the transgenic lines carrying these genes exhibited rachis resistance (Liu *et al*., 2008). Transcriptomic studies involving NILs with QTL‐3BS and QTL‐5A have also identified many differentially expressed genes (Schweiger *et al*., 2013). The RNA‐Seq analysis of Wangshuibai and its FHB susceptible mutant with deletion of the QTL‐Fhb1 region identified several differentially expressed genes but none were selected to have rachis resistance (Xiao *et al*., 2013). Recently, gene expression profiling of NILs containing QTL‐2DL revealed eight candidate genes but only one gene was localized on the 2DL chromosome (Long *et al*., 2015). Although several candidate genes were identified in most of the studies, none could pinpoint and explain the resistance mechanisms. Also, there were only a few attempts towards functional characterization of the genes. The application of new tools could help in understanding the genetic determinants underlying the FHB QTL. The functional characterization of mapped QTL using an alternative approach, such as metabolomics, is considered one of the best tools to decipher the resistance mechanisms and genes underlying FHB resistance (Kushalappa and Gunnaiah, 2013). Such an approach has led to the identification of several RR metabolites in barley (Bollina *et al*., 2010; Kumaraswamy *et al*., 2011) and wheat (Gunnaiah *et al*., 2012). In potato, not only the RR metabolites but also their biosynthetic resistance genes were identified (Pushpa *et al*., 2013; Yogendra *et al*., 2014, 2015).

Hydroxycinnamic acid amides (HCAAs), a class of several complex secondary metabolites produced in the phenylpropanoid pathway, are induced in plants in response to pathogens (Gunnaiah *et al*., 2012; Muroi *et al*., 2009, 2012; von Röpenack *et al*., 1998). HCAAs reduce pathogen advancement through their antimicrobial and cell wall reinforcement properties (Ishihara *et al*., 2008; Keller *et al*., 1996; Miyagawa *et al*., 1995; Schmidt *et al*., 1998). HCAAs such as coumaroylagmatine, coumaroylputrescine, feruloylagmatine and feruloylputrescine were identified as effective defence metabolites in *Arabidopsis thaliana* rosette leaves infected with *Alternaria brassicicola* (Muroi *et al*., 2009). HCAAs are biosynthesized by condensation of hydroxycinnamoyl‐CoA thioesters produced from phenylalanine via the phenylpropanoid pathway with aromatic amines by amine‐specific hydroxycinnamoyl transferases (Edreva *et al*., 2007; Facchini *et al*., 2002).

In this study, we identified several fold change differences in HCAA metabolites including coumaroylagmatine and coumaroylputrescine in FHB QTL‐2DL NIL‐R compared to the susceptible NIL (NIL‐S). The *TaACT* gene responsible for biosynthesizing these high fold‐change metabolites was found within the QTL‐2DL region. The transcript expression, disease severity and fungal biomass as estimated by quantification of relative copy number of a housekeeping fungal gene were also studied. Further, functional characterization of *TaACT* was performed using VIGS in NIL‐R and a complementation study in the Arabidopsis mutant lacking the functional *AtACT* gene.

## Results

### Disease severity in NILs

**Figure 1:**
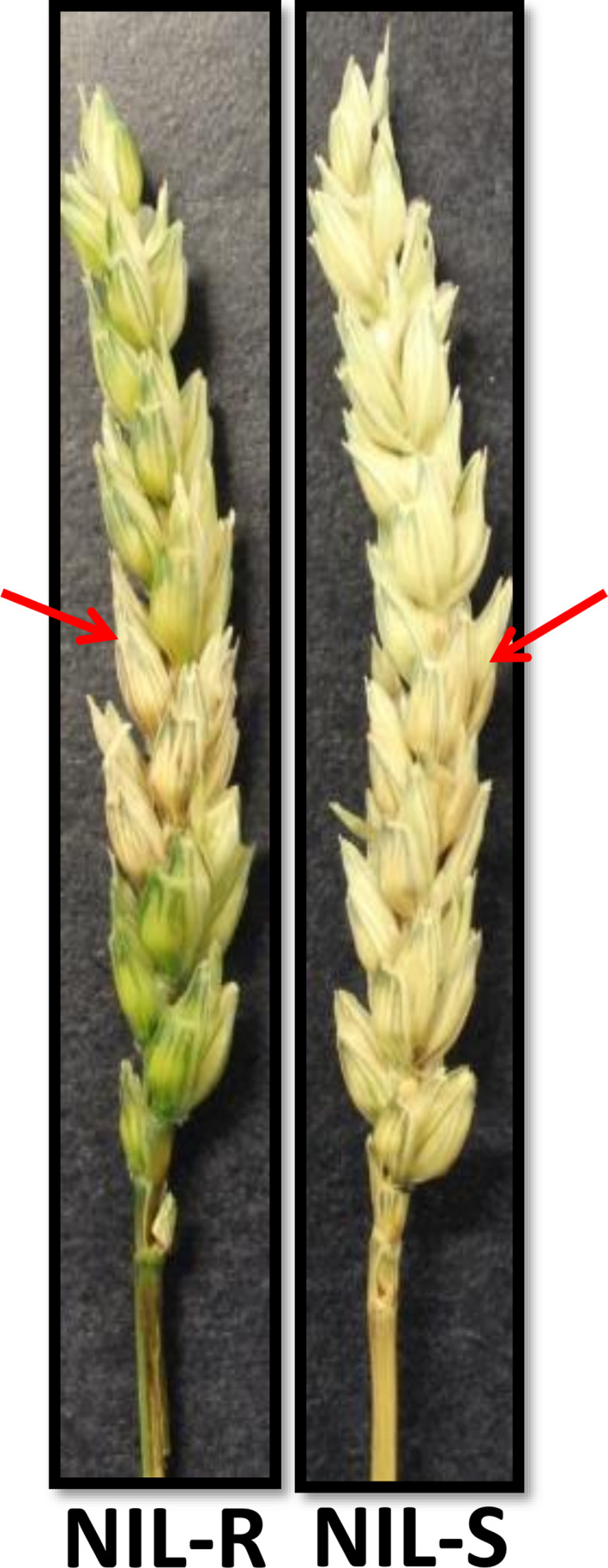
*F. graminearum*‐infected spikes of wheat NILs with resistant and susceptible alleles of QTL‐2DL at 15 dpi. The arrows indicate the spikelets inoculated.

**Figure 2:**
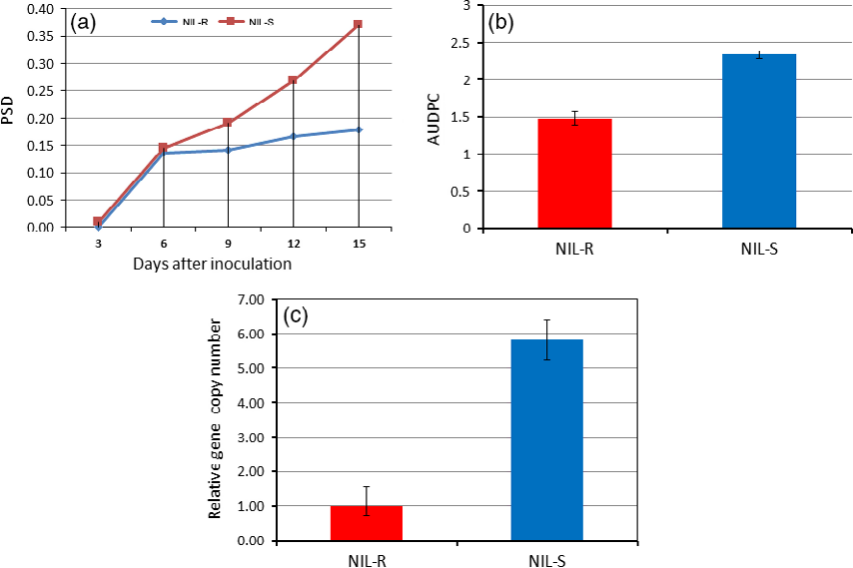
Disease severity and fungal biomass in wheat NILs based on visual observations and RT‐qPCR following point inoculation of *F. graminearum*; (a) proportion of spikelets diseased (PSD); (b) area under the disease progress curve (AUDPC) calculated based on PSD observations taken every three dpi until 15 dpi; (c) fungal biomass (relative gene copy number based on RT‐qPCR) in resistant NIL (NIL‐R) and susceptible NIL (NIL‐S) at seven dpi upon *F. graminearum* inoculation.

The disease severity in spikes of NILs was assessed. Dark brown discoloration due to fungal infection was observed at 3 days postinoculation (dpi) of spikelets in NIL‐S, whereas it was observed only at six dpi in NIL‐R. The uninoculated spikelets above and below the inoculated spikelets in NIL‐S were diseased and bleached at nine dpi. At 15 dpi, almost all of the spikelets were diseased in NIL‐S, whereas only a few were diseased in NIL‐R (Figure 1). The proportion of spikelets diseased (PSD) (Figure 2a) and the area under the disease progress curve (AUDPC) (Figure 2b) at 15 dpi were significantly (*P* < 0.05) lower in the NIL‐R (0.17 and 1.48, respectively) than in NIL‐S (0.36 and 2.33, respectively).

In rachis samples, the fungal biomass, estimated by measuring the relative transcript levels of the *F. graminearum* housekeeping gene *Tri6* (Transcription factor regulating trichothecene biosynthesis) was significantly (*P* < 0.05) lower in NIL‐R than in NIL‐S (Figure 2c), confirming the discrimination of resistance based on disease severity. This indicated that the NIL‐R was significantly more resistant than the NIL‐S.

### Metabolite profiling in NILs

The metabolite profiling of wheat rachis inoculated with *F. graminearum* at 72 h postinoculation (hpi) identified a wide range of metabolites. Among these metabolites, the abundance of two HCAAs, coumaroylagmatine and coumaroylputrescine was 28‐ and 9.5‐fold higher in NIL‐R than in NIL‐S, respectively (Table 1). No other HCAAs were detected.

**Table 1:** Fold change in abundance of resistance‐related (RR) metabolites detected in wheat rachis following *F. graminearum* inoculation

Observed Mass	AME (ppm)	Name	Molecular formula	Retention time (min)	Fold change in NIL‐R relative to NIL‐S	Matched fragmentation patterns	Category	References
276.1592	2.3	Coumaroylagmatine	C14H20N402	12.2	28.7	119.0, 147.0, 218.1, 233.17, 231.3, 258.1, 260.0	HCAAs	Muroi *et al*. (2009); Wen *et al*. (2014); Gorzolka *et al*. (2014)
234.1373	2.2	Coumaroylputrescine	C13H18N202	10.1	9.5	93.1, 119.1, 147.0, 162.2, 191.2, 204.1, 218.1, 233.3	HCAAs	Muroi *et al*. (2009); Moheb *et al*. (2011); Wen *et al*. (2014);

### Histochemical localization of HCAAs

**Figure 3:**
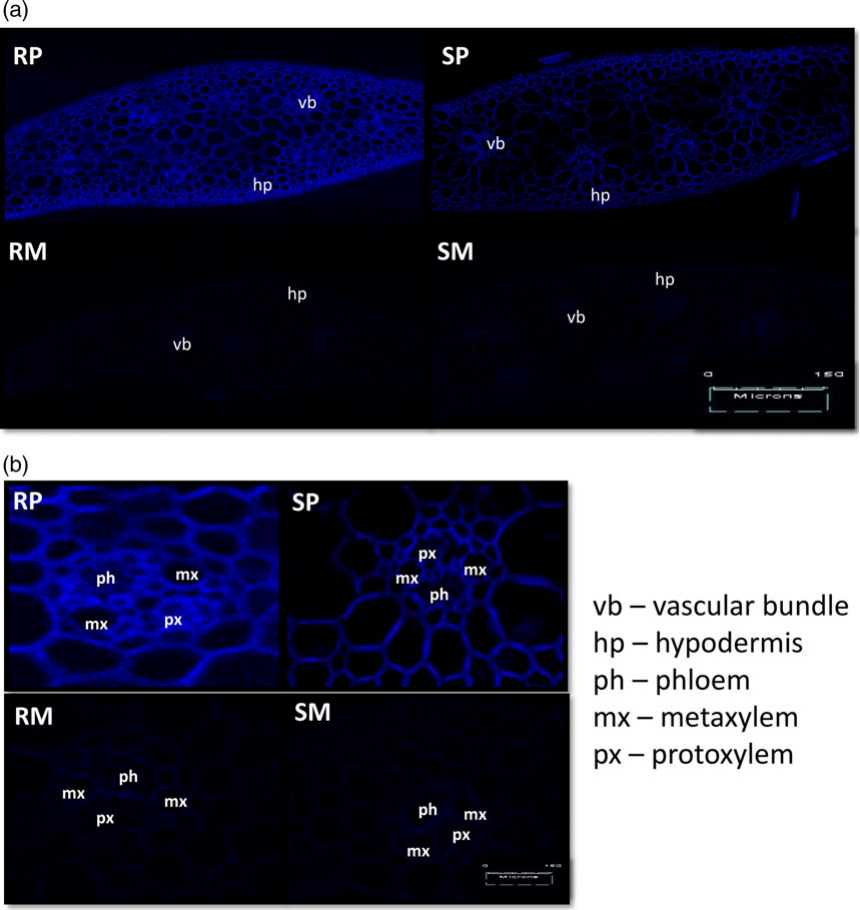
(a) Histochemical localization of HCAAs in rachis cross sections using laser scanning confocal microscopy; (b) histochemical localization of HCAAs in expanded vascular bundles. RP is NIL‐R with *F. graminearum* (pathogen) inoculation, RM is NIL‐R with mock inoculation, SP is NIL‐S with *F. graminearum* inoculation, SM is NIL‐S with mock inoculation. The acronyms are as follows: mx is metaxylem, px is protoxylem, ph is phloem and vb is vascular bundle.

The induction of coumaroylagmatine and coumaroylputrescine in NIL‐R was confirmed by specific staining and fluorescence at specific wave length of cross sections of infected rachis. Stronger chemifluorescence at 405 nm, the typical spectrum of HCAA, was observed in pathogen‐infected NIL‐R cells than in NIL‐S, and also in mock‐treated NIL‐R and NIL‐S (Figure 3). This high chemifloroscence was considered to be mainly due to HCAAs, as we did not find any other high fold‐change phenolics or flavones, which also can be stained with Neu's reagent.

### Identification of candidate gene *TaACT* in the QTL‐2DL

**Figure 4:**
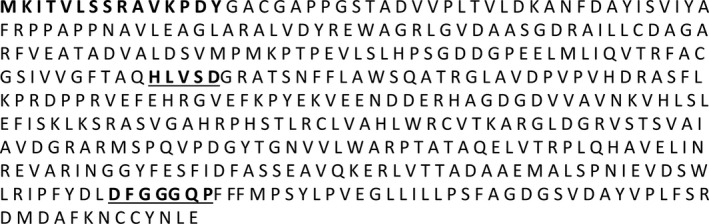
Amino acid sequence of *TaACT*. The two amino acid motifs conserved in the super family are underlined below the sequences, and the N‐terminal‐specific 15 amino acid sequence that is similarly found in most other reported ACTs is shown in bold fonts.

**Figure 5:**
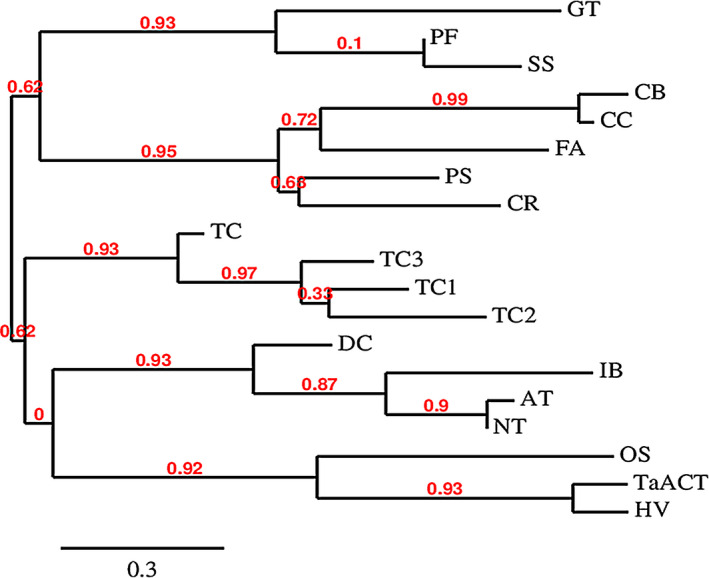
Evolutionary analysis of *TaACT*. Protein sequences used for phylogenetic analysis are as follows: HV = agmatine coumaroyl transferase [*Hordeum vulgare*—AAO73071.1], TaACT = wheat agmatinecoumaroyltransferase (*Triticum aestivum*—KT962210), OS = Putative anthranilate N‐benzoyltransferase [*Oryza sativa Japonica Group*—AAM74310.1], NT = hydroxycinnamoyl transferase [*Nicotiana tabacum*—AJ507825], AT = hydroxycinnamoyl‐CoA shikimate/quinate hydroxycinnamoyl transferase [*Arabidopsis thaliana*—NM_124270], IB = N‐hydroxycinnamoyl/benzoyltransferase [*Ipomoea batatas*—AB035183], DC = anthranilate N‐hydroxycinnamoyl/benzoyltransferase [*Dianthus caryophyllus*—Z84383], TC2 = 2‐debenzoyl‐7,13‐deacetylbaccatin III‐2‐O‐benzoyl transferase [*Taxus cuspidate*= AAG38049.1], TC1 = 10‐deacetylbaccatin III‐10‐O‐acetyl transferase [*Taxus cuspidate*—AAF27621.1], TC3 = taxadienol acetyl transferase [*Taxus cuspidate*—AAF34254.1], TC = 3′‐N‐debenzoyl taxol N‐benzoyltransferase [*Taxus Canadensis*—AAM75818.1], CR = deacetyl vindoline 4‐O‐acetyltransferase [*Catharanthus roseus*—AAC99311.1], PS = salutaridinol 7‐O‐acetyltransferase [*Papaver somniferum*—AAK73661.1], FA—alcohol acyltransferase [*Fragaria ananassa*—AAG13130.1], CC = acetyl‐CoA:benzyl alcohol acetyltranferase [*Clarkia concinna*—AAF04784.1], CB = acetyl‐CoA: benzyl alcohol acetyltransferase [*Clarkia breweri*—AAC18062.1], SS = malonylCoA:anthocyanin 5‐O‐glucoside‐6‴‐O‐malonyl transferase [*Salvia splendens*—AAL50566.1], PF = malonylCoA:anthocyanin 5‐O‐glucoside‐6‴‐O‐malonyl transferase [*Perilla frutescens*—AAL50565.1], GT = Anthocyanin 5‐aromatic acyltransferase [*Gentiana triflora*—BAA74428.1].

The two HCAA candidate metabolites identified here were mapped onto metabolic pathways to identify their biosynthetic enzymes. Agmatinecoumaroyl transferase (ACT) is a rate‐limiting enzyme involved in the biosynthesis of these metabolites (Burhenne *et al*., 2003; Muroi *et al*., 2009, 2012). BLAST analysis positioned *TaACT* as the closest gene match for this enzyme within the presumed QTL interval on 2DL. The *TaACT* full‐length gene, including 546‐bp promoter region was sequenced using the genomic DNA from both resistant and susceptible NILs. Analyses of *TaACT* using both the genomic DNA and cDNA determined the *TaACT* to be of 1326 bp in length and were devoid of introns. Sequence comparison of *TaACT* from NIL‐R and *T. aestivum* cv. Chinese spring showed that the gene sequence is highly conserved. Comparison of the DNA sequences between NIL‐R and NIL‐S revealed two inversions (2 bp) and 67 SNPs (Figs. S1 & S2). The conserved domain analysis of the encoded protein using the MOTIF Search tool (http://www.genome.jp/tools/motif/), revealed the presence of a transferase domain. The predicted protein consisted of two consensus motifs (Figure 4): (i) the HLVSD motif that starts at His‐153 and is identical to the HXXXD motif that is commonly found in the transferase family which are responsible for CoA‐dependent acyl transfer (St‐Pierre *et al*., 1998) and, (ii) the DFGGGQP motif, that starts at residue Asp‐387, which is a motif of unknown function (Burhenne *et al*., 2003). This protein also has a similar N‐terminal‐specific 15 amino acid sequence MKITVLSSRAVKPDY that is found in most other reported *ACT*s. BLASTP search of *TaACT* in UniProt database indicated 83% identity with barley ACT (*HvACT*) protein. Phylogenetic analysis further demonstrated a close relatedness of these orthologs (Figure 5). It revealed that plant acyltransferases form five evolutionary groups (Burhenne *et al*., 2003), and *HvACT* falls under the fifth group. This group defines plant acyltransferases that are involved in transferring acyl groups to the acceptors. For further confirmation of predicted protein size (~48 kDa), the *TaACT* was heterologously expressed in *E. coli* (BL21) cells. The protein extract from supernatant was used to run the SDS‐PAGE. A protein band with molecular weight approximately 48 kDa was observed (Fig. S3), and it was comparable to the previously reported *HvACT* (Burhenne *et al*., 2003). Our results confirmed the presence of *ACT* gene within the QTL region on 2DL, thus suggesting a possible link of this gene to the induction of high levels of coumaroylagmatine and coumaroylputrescine following pathogen inoculation.

### Expression of gene *TaACT* based on semi‐quantitative PCR (semi‐qPCR) and real‐time quantitative PCR (RT‐qPCR)

**Figure 6:**
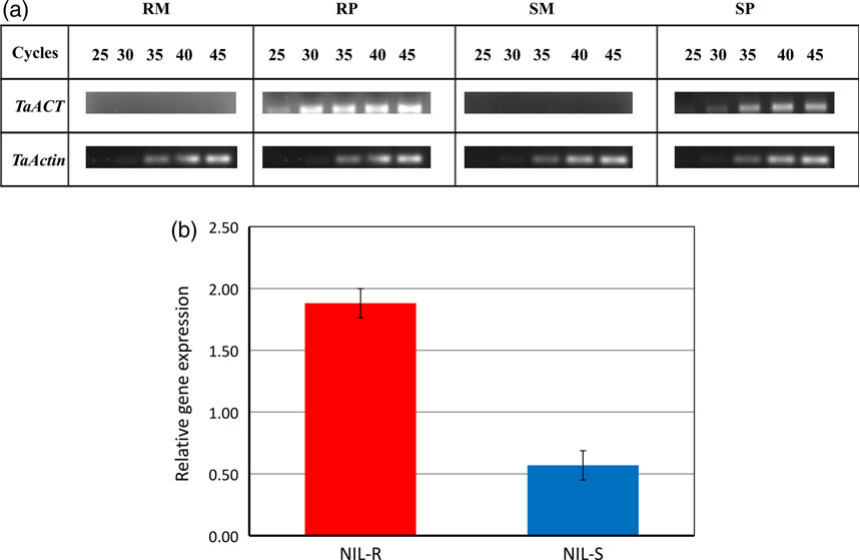
Gene expression analysis of *TaACT* gene in wheat rachis at 72 hpi, (a) gene expression of *TaACT* based on Semi‐qPCR; (b) gene expression of *TaACT* in pathogen inoculated treatments (RP, SP; the mock inoculated did not show any expression, thus not presented) based RT‐qPCR. *TaActin* gene was used as internal standard. RM = resistant mock, RP = resistant pathogen, SM = susceptible mock, SP = susceptible pathogen treatments.

The expression of the gene *TaACT* was examined using semi‐qPCR and RT‐qPCR. Although semi‐qPCR did not reveal any expression in mock‐treated samples from NIL‐R and NIL‐S, at 72 hpi, a high expression was observed in NIL‐R relative to NIL‐S upon pathogen inoculation (Figure 6a). This suggested that the gene *TaACT* was induced in wheat rachis only after pathogen inoculation. A semi‐qPCR of an increased template (cDNA) quantity from 20 to 100 ng revealed the difference (data not shown), and this was further confirmed by RT‐qPCR, which indicated the expression to be 3.2‐fold higher in NIL‐R than in NIL‐S (*P* < 0.05) (Figure 6b).

### Functional characterization of *TaACT* using VIGS

**Figure 7:**
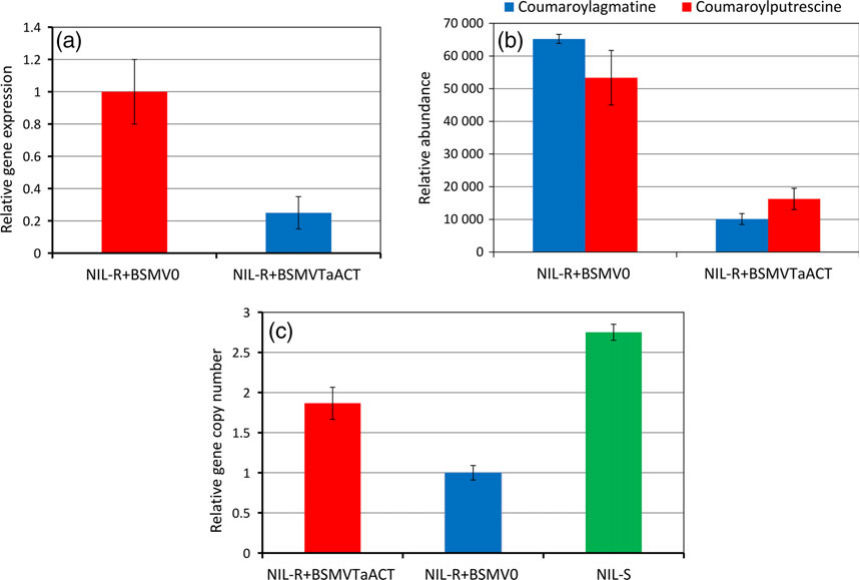
Effect of *TaACT* silencing in NIL‐R inoculated with *F. graminearum* or mock solution inoculation: (a) relative transcript expression of *TaACT*; (b) relative metabolite abundances of coumaroylagmatine and coumaroylputrescine in silenced (NIL‐R+BSMV_*T*_ _*a*_ _*ACT*_ ) and nonsilenced (NIL‐R+BSMV_0_) NIL‐R; (c) biomass (as relative gene copy number based on RT‐qPCR) of *F. graminearum* in wheat rachis of silenced (NIL‐R+BSMV_*T*_ _*a*_ _*ACT*_ ), nonsilenced (NIL‐R+BSMV_0_) NIL‐R and NIL‐S.

At 72 hpi of *F. graminearum*, the silenced plants NIL‐R+BSMV_*TaACT*_ (BSMV (Barley Stripe Mosaic Virus) + *TaACT* insert) showed significant (*P* < 0.05) (74.29%) reduction in *TaACT* transcript abundance as compared to nonsilenced plants NIL‐R+BSMV_0_ (BSMV + without *TaACT* insert) confirming the silencing of the target gene in wheat rachis (Figure 7a). Metabolite analysis revealed that the relative abundance of coumaroylagmatine and coumaroylputrescine was significantly reduced in plants NIL‐R+BSMV_*TaACT*_ compared to NIL‐R+BSMV_0_ plants by 6.4‐ and 3.2‐fold respectively (Figure 7b). The fungal biomass in NIL‐R+BSMV_*TaACT*_ was significantly higher than in NIL‐R+BSMV_0_ (1.86 folds, *P* < 0.05) (Figure 7c). Whereas the fungal biomass in NIL‐R+BSMV_*TaACT*_ was 1.47‐fold lower than in NIL‐S, indicating possible involvement of other resistance genes associated with the QTL‐2DL.

### Functional characterization of *TaACT* based on complementation study in Arabidopsis

**Figure 8:**
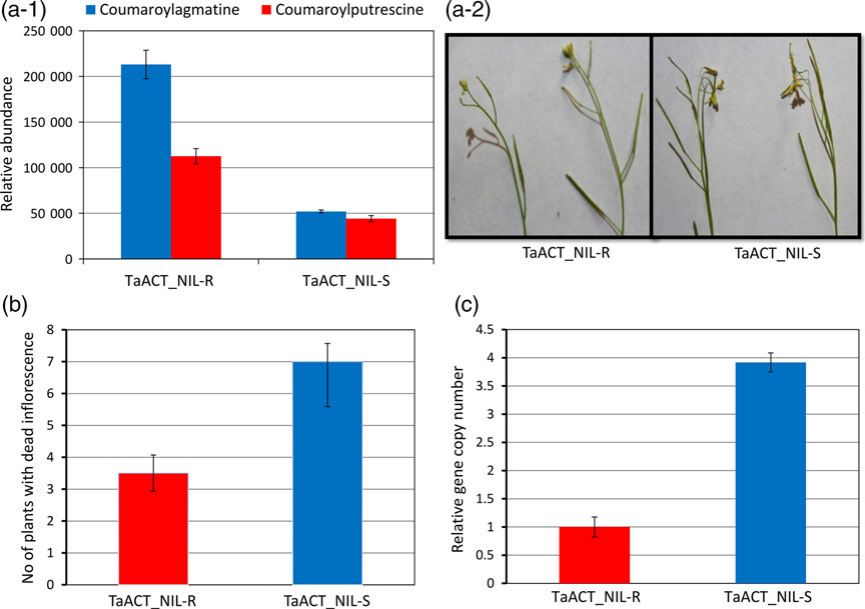
Effect of *TaACT* overexpressing Arabidopsis plants on resistance to *F. graminearum*. (a‐1) Relative metabolite abundances of coumaroylagmatine and coumaroylputrescine in NIL‐R (TaACT_NIL‐R) and NIL‐S (TaACT_NIL‐S) *TaACT* expressing Arabidopsis plants; (a‐2) symptoms observed in Arabidopsis inflorescence (diseased flowers) expressing *TaACT* gene from NIL‐R (TaACT_ NIL‐R) and NIL‐S (TaACT_ NIL‐S); (b) the disease incidence in transgenic Arabidopsis plants inoculated with *F. graminearum*, quantified as number of plants with inflorescence diseased (of 25 per replicate, in four replicates); c) biomass (relative gene copy number based on RT‐qPCR) of *F. graminearum in* NIL‐R (TaACT_NIL‐R) and NIL‐S (TaACT_NIL‐S) *TaACT* expressing Arabidopsis transgenic plants.

Metabolite profiling of the inflorescence of Arabidopsis plants revealed significantly (*P* < 0.05) higher abundances of coumaroylagmatine (4.1‐fold) and coumaroylputrescine (2.5‐fold) in plants over‐expressing the *TaACT* from NIL‐R (*TaACT_*NIL‐R) than plants over‐expressing NIL‐S (*TaACT_*NIL‐S) (Figure 8a‐1). Further, the inflorescence of transgenic Arabidopsis in plants was inoculated with *F. graminearum* and the number of plants with inflorescence diseased was assessed at six dpi (Figure 8a‐2). The mean number of plants with diseased inflorescence of 25 plants and for four replicates was significantly (*P* < 0.01) reduced in plants over‐expressing NIL‐R *TaACT* than in plants over‐expressing NIL‐S *TaACT* (Figure 8b). The fungal biomass reduction in inflorescence of Arabidopsis also followed a same trend. The fungal biomass was 3.8‐fold higher in *TaACT_*NIL‐S than in *TaACT_*NIL‐R over‐expressing plants (Figure 8c). This suggests that some of the inversions and SNPs found in NIL‐S were responsible for the reduced functionality and resistance. Although the *TaACT* gene from NIL‐R can be used to replace nonfunctional genes in commercial wheat cultivars based on genome editing, the use of specific nucleotide replacement requires further studies to validate the functions of these SNPs.

## Discussion

The present study reports an integrated approach of metabolomics, gene sequencing information, gene expression histochemical studies and heterologous protein expression to identify and confirm the presence of the candidate gene *TaACT* in the FHB QTL‐2DL region. Further, for the first time in wheat our study reports the functional characterization of *TaACT* through VIGS and a complementation study in Arabidopsis. Taken together, our results revealed the presence of the candidate gene *TaACT* in FHB QTL‐2DL in NIL‐R and its resistance functions against FHB. Its role in resistance and its application in breeding are discussed.

### FHB resistance in NIL‐R is due to high fold induction of HCAAs

Resistance in plants is controlled by hierarchies of resistance (*R*) genes, including regulatory genes and the regulated genes that biosynthesize RR metabolites and proteins, which directly suppress the pathogen development through their antimicrobial and cell wall reinforcement properties (Kushalappa *et al*., 2016a). Resistance in wheat to the spread of *F. graminearum* through rachis is mainly due to the production of phytoalexins that are antimicrobial, and/or to the deposition of phenylpropanoids to reinforce the cell wall, thus preventing further progress of the pathogen in plant (Gunnaiah *et al*., 2012). Coumaroylagmatine and coumaroylputrescine types of HCAAs were induced in high fold‐change in resistant NIL. The reinforcement of cell walls by these compounds was confirmed by histochemical localization of HCAAs. Deposition of feruloyl‐3′‐methoxytyramine, feruloyltyramine and *p‐*coumaroyltyramine has been reported from onion cells at the infection sites, following inoculation with *Botrytis allii* (Ishihara *et al*., 2008). Different HCAAs including coumaroylagmatine and coumaroylputrescine were proved to be antimicrobial and highly induced in *Arabidopsis thaliana* rosette leaves infected with *Alternaria brassicicola* (Muroi *et al*., 2009) and in transgenic torentia expressing *AtACT* to resist *Botrytis cinerea,* and arthropod herbivores (Muroi *et al*., 2012). Accumulation of coumaroylagmatine and its antimicrobial effect was reported in barley leaves infected with *Erysiphe graminis* f. sp. *hordei* (von Röpenack *et al*., 1998). HCAAs such as *p*‐coumaroyl serotonin and feruloyl serotonin were detected in rice leaves infected with *Bipolaris oryzae* (McLusky *et al*., 1999). Several HCAAs, such as feruloylputrescine, *p*‐coumaroyltyramine, N‐feruloyltyramine, 4‐coumaroyl‐3‐hydroxy agmatine, feruloylagmatine, coumaroylagmatine, terrestriamide and feruloylserotonin, were induced in potato leaves against *Phytophthora infestans* (Keller *et al*., 1996; Pushpa *et al*., 2013; Yogendra *et al*., 2014). In our study, the cell wall reinforcement in NIL‐R was also associated with lower disease severity and fungal biomass relative to NIL‐S. Accordingly, our results suggest that coumaroylagmatine and coumaroylputrescine are responsible for resistance in QTL‐2DL against the spread of *F. graminearum* through rachis.

### *TaACT* induced high levels of coumaroylagmatine and coumaroylputrescine

The coumaroylagmatine and coumaroylputrescine responsible for resistance were searched in metabolic pathway networks to identify their respective biosynthesizing enzymes and genes. This led to the identification of the *ACT* gene, which is also known to catalyse the last step of biosynthesis of these two HCAAs (Burhenne *et al*., 2003; Muroi *et al*., 2009, 2012). We temporarily located the FHB QTL‐2DL on wheat 2DL chromosome and the gene encoding functional *TaACT* within the QTL‐2DL region. This gene was sequenced and confirmed by comparing with the sequences and conserved protein domains of the previously characterized *HvACT* gene (Burhenne *et al*., 2003). Closest phylogenetic relationship with *HvACT* confirmed *TaACT* is a member of the group five acyltransferases, which is involved in transferring acyl groups to the acceptors. Further, the recombinant protein was expressed in *E. coli,* and the protein size was similar to the protein size of barley *HvACT*. In barley and wheat, the coexistence of hydroxycinnamoyl agmatines and putrescines has been demonstrated, and they are induced by the same biological stimuli (Fester *et al*., 1999; Ogura *et al*., 2001). Our report here further confirms that the *TaACT* in wheat QTL‐2DL is responsible for the production of high abundance of coumaroylagmatine and coumaroylputrescine. In addition, based on semi‐qPCR and qRT‐PCR studies, it was confirmed that *TaACT* was expressed only after pathogen invasion. Similarly in Arabidopsis, the *AtACT* gene was highly expressed after pathogen inoculation (Muroi *et al*., 2009).

### Functional validation of *TaACT*

To further assess the effect of *TaACT* on resistance to spread of *F. graminearum* through rachis, it was silenced in NIL‐R. The transcript abundance of *TaACT* and its respective metabolites coumaroylagmatine and coumaroylputrescine abundances were significantly reduced after silencing. The fungal biomass increased in silenced NIL‐R relative to nonsilenced. In the same way, transient gene silencing of MYB10 decreased the flavonoid/phenylpropanoid metabolism in strawberry (Medina‐Puche *et al*., 2014). Silencing of hydroxycinnamoyl‐CoA:hydroxycinnamoyl transferase gene in *N. benthamiana* stems inhibited the accumulation of a lignin polymer, dimethoxylated syringyl, affecting the cell wall reinforcement (Hoffmann *et al*., 2004). Similarly, the virus‐induced silencing of *StWRKY1* significantly reduced the abundance of N‐feruloyltyramine, N‐feruloyloctopamine, feruloylputrescine and feruloylagmatine types of HCAAs compromising late blight resistance by reducing reinforcement of secondary cell walls in potato (Yogendra *et al*., 2015). Knock‐down of *FcWRKY70* in kumquat down‐regulated *ADC* (arginine decarboxylase) abundance and decreased putrescine level accompanied by compromised dehydration tolerance (Gong *et al*., 2015).

The *TaACT* gene function was also proved based on a complementation study using *act* mutant Arabidopsis lines which lacked the ability to biosynthesize coumaroylagmatine and coumaroylputrescine. Mutant Arabidopsis plants overexpressing the *TaACT* from NIL‐R had higher abundances of coumaroylagmatine and coumaroylputrescine metabolites as compared to plants overexpressing *TaACT* from NIL‐S. In addition, the former also resulted in decreased disease severity and amount of fungal biomass, thus confirming that this difference is mainly due to polymorphic sequences of *TaACT*. Model plants have been used to prove the functions of several genes. Transgenic *A. thaliana* expressing a barley UDP‐glucosyltransferase exhibited resistance to the mycotoxin deoxynivalenol (Shin *et al*., 2012). Transgenic expression of polygalacturonase‐inhibiting proteins in Arabidopsis and wheat increased the resistance to flower pathogen *F. graminearum* (Ferrari *et al*., 2012). Also, overexpression of *PvPGIP2* was shown to be effective against a wheat foliar pathogen, *B. sorokiniana* due to increased polygalacturonase‐inhibiting proteins (Janni *et al*., 2008). Taken together, these results provide compelling evidence and support that *TaACT* is one of the candidate genes responsible for FHB resistance in wheat QTL‐2DL, through deposition of HCAAs to reinforce secondary cell walls, thus preventing further spread of pathogen throughout rachis.

In conclusion, we have identified here the *TaACT* gene located in wheat QTL‐2DL and confirmed its resistance function against FHB under greenhouse conditions. The FHB resistance of the *TaACT* should also be effective under field conditions. Stacking of a few genes with similar FHB resistance effects should significantly reduce FHB under field conditions. The *TaACT* therefore can be used in FHB resistance breeding programmes either through development of genic markers (Kage *et al*., 2015) or through replacement of a nonfunctional *Taact* gene in commercial cultivars with functional *TaACT* genes based on genome editing (Kushalappa *et al*., 2016b). Pyramiding of a few genes should result in a resistant cultivar that reduces application of fungicide.

## Experimental procedures

### Plant material and experimental layout

The NILs carrying resistant and susceptible alleles of QTL‐2DL were derived from a cross BW301 × HC374 (McCartney *et al*., 2007). The BW301 is a FHB susceptible hard red spring wheat line from Western Canada, and HC374 is a resistant line derived from Wuhan‐1. The experiment was laid out in a randomized complete block design (RCBD) with two genotypes (resistant and susceptible NILs), two inoculations (pathogen and mock solution) and five replications over time to include sufficient block effect. Initially, five seeds were planted per pot, and later each pot was maintained with only three plants. The plants were grown in the greenhouse which was maintained at 23 ± 2 °C with 16 h of light and 8 h of dark.

### Pathogen production and inoculation

An isolate of *F. graminearum* (GZ‐3639, obtained from Dr. R.H. Proctor, USDA) was grown on potato dextrose agar (PDA) plates and incubated at 26 °C for 4 days. For spore production, *F. graminearum* was further subcultured on Rye B agar media and kept inverted by exposing the plates to near UV light for a period of 3 days. Macroconidia were harvested from 7‐day‐old cultures, spore concentration was determined using a haemocytometer (American Scientific, Ohio, USA), and the final concentration was adjusted to 1 × 10^5^ macroconidia/mL (Chamarthi *et al*., 2014). At 50% anthesis (GS = 65), three alternate spikelet pairs of ten spikes per replicate were individually inoculated with 10 μL of fungal spore suspension containing of 1 × 10^5^ macroconidia/mL or mock solution (water) using a syringe with an autodispenser (GASTIGHT 1750DAD, Reno). Inoculated plants were covered with water‐sprayed polythene bags to maintain high moisture content. At 48 hpi, the bags were removed.

### Sample collection, metabolite extraction and metabolite analysis using liquid chromatography high‐resolution mass spectrometry (LC‐HRMS)

At 72 hpi, ten inoculated spikes per replicate were harvested. The spike region, with three inoculated and three uninoculated (a total of six) pairs of spikelets was retained. Spikelets and rachis samples were separately collected, immediately frozen in liquid nitrogen and stored at −80 °C until further use. Of ten, five rachis samples (remaining five were used for histochemical analysis) were ground in liquid nitrogen, and the metabolites were extracted in 60% ice‐cold aqueous methanol. The extract was sonicated for 15 min at 25 °C, centrifuged, the supernatant was filtered and then 5 μL of the clear sample extract was used for metabolite analysis using LC‐HRMS (at IRCM, Montreal, Canada), by following the previously established protocol (Bollina *et al*., 2010).

### Data processing using MZmine software and statistical analysis

The output data Xcaliber RAW files from LC‐HRMS were converted into mzXML format. Converted files were imported to bioinformatics tool MZmine2 for peak de‐convolution, peak detection and spectral filtering (Katajamaa *et al*., 2006). The observed monoisotopic masses (negative ionization) and their respective abundances (relative intensity) were imported to MS Excel. The relative peak intensities of monoisotopic masses of metabolites were subjected to Student's *t‐*test (SAS v 9.3) in pairwise treatment combinations (RP vs RM, RM vs SM, SP vs SM and RP vs SP, where RP = resistant NIL inoculated with pathogen, RM = resistant NIL inoculated with mock solution, SP = susceptible NIL inoculated with pathogen, SM = susceptible NIL inoculated with mock solution). Treatment significant metabolites with *P *<* *0.05 were retained for further analysis. The metabolites significantly higher in abundance in resistant than susceptible NIL were considered as RR metabolites. Further, these metabolites were grouped into RR constitutive (RRC = RM > SM) and RR induced (RRI = (RP > RM) >(SP >SM)) metabolites. The fold‐change in abundance of metabolites in NIL‐R was calculated relative to NIL‐S (NIL‐R/NIL‐S) (Gunnaiah *et al*., 2012). Only the selected high fold‐change RRI metabolites were prioritized for further candidate gene identification.

### Putative identification of metabolites

The RR metabolites were putatively identified based on three criteria: (i) accurate mass match with the metabolites reported in several databases such as PlantCyc, METLIN, KNApSAcK, LIPIDMAPS, NIST and KEGG, with an accurate mass error, AME ≤ 5 ppm (Kushalappa and Gunnaiah, 2013; Tohge and Fernie, 2010); (ii) fragmentation pattern mass match with databases and literature (Matsuda *et al*., 2009); (iii) *in silico* confirmation of fragmentation based on Masspec scissor in ChemSketch (ACD labs, Toronto) (Matsuda *et al*., 2009).

### Disease severity and quantification of fungal biomass

To evaluate rachis resistance in wheat genotypes, two NILs with resistant and susceptible alleles were planted in RCBD with three biological replications at 3‐day intervals to include sufficient block effect. At the 50% anthesis (GS = 65), a single pair of spikelets in the middle of the spike with five spikes per replicate was point inoculated with 10 μL of spore suspension (1 × 10^5^ macroconidia/mL). The number of diseased spikelets was recorded at 3‐day intervals until 15 dpi. Spikelets with both dark brown discoloration and bleaching symptoms were considered as diseased. From these data, the PSD (proportion of spikelets diseased = number of spikelets diseased/total number of spikelets in spike) and the AUDPC were calculated (Hamzehzarghani *et al*., 2008). The PSD among observations was analysed for significance based on ANOVA using SAS program (SAS v 9.3).

To quantify the fungal biomass, a separate experiment was conducted with two treatments (NIL‐R and NIL‐S) and three biological replications over time. At the 50% anthesis (GS = 65), three alternate pairs of spikelets in the middle of the spike with five spikes per replicate was point inoculated with 10 μL of spore suspension (1 × 10^5^ macroconidia/mL). Samples were collected at six dpi, and the fungal biomass was quantified in rachis samples as relative gene copy number of the fungal housekeeping gene *Tri6*. Genomic DNA was isolated from rachis samples using a DNeasy Plant Mini Kit (Qiagen, Canada), and DNA quality was assessed by gel electrophores in 1% agarose gel and quantified by nano‐drop (Thermo‐Scientific Canada). Equal quantities of DNA (20 ng) were used for the relative fungal biomass quantification in the NILs under study. RT‐qPCR was performed using fungal‐specific gene (*Tri6*) primers. The abundance of *Tri6* gene was normalized with *TaActin,* and the results obtained from RT‐qPCR experiment were used to estimate the fungal biomass (Kumar *et al*., 2015).

### Physical localization of QTL‐2DL and identification of *TaACT* gene

The presence of SSR markers, WMC245, GPW8003, GWM539 and GWM608 was used to define the interval for QTL‐2DL. Sequence of WMC245 was retrieved from available GrainGenes database, and markers GPW8003, GWM539 and GWM608 were sequenced in our laboratory. We directly sequenced the PCR products and subjected to BLASTN (Altschul *et al*., 1990) search on the international wheat genome‐sequencing consortium (IWGSC) wheat genome database for temporary physical localization of the QTL (we considered only the best 2DL BLAST hits). High fold change RRI metabolites were mapped in metabolic pathways to identify the catalytic enzymes and their coding genes. Sequences of these genes were searched by BLAST against IWGSC database to confirm their colocalization within the predicted QTL‐2DL region. Contigs identified as the best hits were retrieved from the database, and gene prediction was performed using the SoftBerry's FGENESH program (Solovyev *et al*., 2006) to study gene structure. The identified gene was amplified using gene‐specific primers designed using the NCBI Primer‐BLAST tool (Ye *et al*., 2012).

### Cloning, sequencing and sequence analysis of *TaACT* gene

Genomic DNA was isolated from the rachis samples of NILs using DNeasy Plant Mini Kit (Qiagen, Canada) and used for the amplification of full‐length *TaACT* gene using gene‐specific primers. PCR was performed as follows: initial denaturation at 95 °C for 5 min, followed by 35 cycles of 94 °C for 30 s, 55 °C for 1 min, 72 °C for 2 min followed by a final extension at 72 °C for 10 min. PCR products were separated on a 1% agarose gel. A band size corresponding to ~1350 bp was then purified from the gel, cloned into the pGEM‐T Easy vector (Promega, Canada) and Sanger sequenced (Genome Quebec, McGill University). The sequence of *TaACT* (Accession No. KT962210) was deposited to the NCBI database. The sequences from both the NILs were aligned to identify sequence variations at the genic region using MultAlin (Corpet, 1988). MOTIF Search tool (http://www.genome.jp/tools/motif/) was used to see the presence of conserved domains in translated amino acid sequences. The Phylogey.fr (http://www.phylogeny.fr/) program was used to perform a multiple sequence alignment and to construct the phylogenetic tree.

### RNA isolation and candidate gene expression based on semi‐qPCR and RT‐qPCR

Total plant RNA was isolated from five biological replicates using RNeasy plant mini kit (Qiagen, Canada). Purified total RNA (1–2 μg) was used to reverse transcribe RNA into cDNA using the AffinityScript cDNA synthesis kit (Agilent Technologies, Canada). The level of expression was determined for *TaACT* gene using gene‐specific primers by semi‐qPCR at an increasing number of cycles (at 25, 30, 35, 40 and 45 cycles) in three biological replicates. The following PCR conditions were used: initial denaturation at 95 °C for 5 min followed by 36 cycles: 1 min of denaturation at 95 °C, 40 s of annealing at 53 °C and followed by a final extension of 1 min at 72 °C. RT‐qPCR was performed using Qi SYBR Green supermix (Bio‐Rad, Canada) in a CFX384^™^ real‐time PCR system (Bio‐Rad, Canada) in five biological replicates. The gene transcript level was normalized to the *TaActin* transcript levels. PCR results were analysed using comparative 2^−ΔΔCT^ method (Livak and Schmittgen, 2001).

### Histochemical localization of HCAAs

The five rachis samples in the region of inoculated spikelets, of ten collected at 72 hpi in the metabolomics study were stored at −20 °C for further use. For cryosectioning, the samples were prepared by embedding tissues in cryomoulds containing Shandon CRYOMATRIX (Richard‐Allan Scientific, Kalamazoo). Cryosectioning (15 μm) was carried out using a cryotome (Leica, CM1850, Canada) machine at −20 °C, and the cross sections were collected on slides coated with 5% 3‐aminopropyltriethoxy‐silane (APES) solution. Sections were washed with distilled water and stained with Neu's reagent (1% 2‐amino ethyl diphenylborinate (Sigma Aldrich, Canada) prepared in absolute methanol) for 5 min and mounted in 15% glycerol. The stained samples were observed under a confocal microscope (Nikon, Eclipse E800, USA) for HCAA chemifluorescence, with excitation at 405 nm with emission filter HQ442/45.

### Expression and purification of recombinant protein in *E. coli*

The coding region of *TaACT* gene was amplified from cDNA using primers containing *EcoRI* and *BglII* restriction sites. PCR product was digested with *EcoRI* and *BglII*, the fragment was ligated into the pTRC‐HisB vector (Invitrogen, Canada). Recombinant vector and empty vector without cloned gene were transformed into *E. coli* BL21 cells and grown in Luria Bertani medium at 28 °C to an optical density of 0.6 at 600 nm (A_600_). The induction of expression was carried out at 18 °C for 15 h by the addition of isopropyl‐1‐thio‐D‐galactopyranoside (IPTG) to a final concentration of 1 mm. After 15 h, 1 mL sample from both recombinant and empty suspension was collected, centrifuged at 10 000 ***g***, processed further and the expression was confirmed on 12% sodium dodecyl sulphate‐polyacrylamide gel electrophoresis (SDS‐PAGE). Remaining cells were immediately pelleted down by centrifugation at 4 °C. Cell pellet was resuspended in 1X LEW buffer (50 mm NaH_2_PO_4_, 300 mM NaCl, pH 8.0), and cells were lysed by adding lysozyme (1 mg/mL), incubating at 4 °C for 1 h, followed by sonication. The supernatant was collected after centrifugation at 12 000 ***g*** for 20 min at 4 °C, and purification was achieved using nickel nitrilotriacetic acid (Ni‐NTA) column (Affymetrix, Canada). Purified protein fraction was detected by Coomassie Brilliant Blue Staining after electrophoresis in 12% SDS‐PAGE. The average protein size of *TaACT* was predicted using ExPAsy‐Compute pI/Mw tool (http://web.expasy.org/compute_pi/).

### Virus‐induced gene silencing of *TaACT*

The PCR product amplified from cDNA using VIGS primers (listed in Table S1) was used to construct the silencing vector. The VIGS primers were designed using the NCBI Primer‐BLAST tool (Ye *et al*., 2012). A 271‐bp fragment of the *TaACT* gene with efficient siRNA generation and no off target genes in the modified viral genome using siRNA Scan tool (http://bioinfo2.noble.org/RNAiScan.htm) and a BLAST search of fragment against GenBank database was chosen. The PCR product was cloned into the pGEM‐T Easy Vector (Promega Corp., WI), confirmed by sequencing and excised using *NotI* (New England Biolabs, MA, USA), thereby generating *NotI* ends. These fragments were subsequently cloned in the *NotI* site of pSL038‐1, a plasmid encoding a modified BSMV γ‐genome segment with a cloning site downstream of the γb gene (Cakir and Scofield, 2008). Clones containing the fragments in the γ‐vector were sequenced to confirm their identity and subsequently used for gene silencing.

The procedures for *in vitro* transcription of α‐, β‐ and γ‐RNAs of the BSMV genome were the same as described by Scofield *et al*. (2012). *In vitro* synthesized BSMV RNAs were three times rub inoculated on both flag leaf and spikes at growth stage 50–55 (Zadoks *et al*., 1974) with a solution containing 1 : 1 : 1 μL (α‐, β‐ and γ‐RNAs) + 22.5 μL of abrasive FES buffer (1% sodium pyrophosphate, 1% bentonite, 1% celite in 0.1 m glycine, 0.06 m dipotassium phosphate). Ten spikes per replicate were infected with each of BSMV + *TaACT* insert and BSMV:0 respectively with five biological replicates. Construct BSMV:0 and BSMV + PDS insert served as negative and positive controls respectively. An experiment involving VIGS resulted in photo‐bleaching symptoms in the wheat spikes 12 days after the viral inoculation (Fig. S4). At 13 days postviral infection, ten spikes each in the negative control and test treatment were inoculated with 10 μL of *F. graminearum* spore suspension (1 × 10^5^ macroconidia/mL) and covered with plastic bags to maintain high humidity. At 48 hpi with *F. graminearum,* plastic covers were removed and samples were collected from five spikes at 72 hpi for metabolite analysis and RT‐qPCR to measure the transcript abundance of *TaACT*. Similarly, total DNA isolated from six dpi samples collected from remaining five spikes was used in the fungal biomass study. The fungal biomass was quantified in NIL‐R+BSMV_0_, NIL‐R+BSMV_*TaACT*_ and NIL‐S.

### *TaACT* functional complementation study in Arabidopsis

The *TaACT* alleles from NIL‐R and NIL‐S were overexpressed in an Arabidopsis *Atact* mutant (At5g61160) background (obtained from TAIR) for their FHB resistance function validation. Homozygous T1 lines for four transgenic events were identified by examining the segregation for hygromycin resistance. Progenies derived from these transgenic events were 100% hygromycin resistant, and homozygous T2 lines derived from these were used for further testing. Samples were collected from 6‐week‐old plants for targeted metabolite analysis with three biological replicates. The detailed procedure followed for development of transgenic plants is given in supplementary data (Procedure S1).

A set of 25 T2 plants each with *TaACT* of NIL‐R or NIL‐S in four replicates over time were inoculated with *F. graminearum* at flowering stage to determine the effect of *F. graminearum* infection. The open flowers of 6‐week‐old plants were inoculated by placing 10 μL droplets of spore suspension (1 × 10^5^ macroconidia/mL). Inoculated plants were covered with plastic bags for 3 days to maintain high humidity, and the symptom development was monitored. The inoculated flowers were diseased in four dpi, and by six dpi, the disease spread to uninoculated flowers within the inflorescence. At six dpi, the number of plants with diseased (dead) inflorescence was assessed (Figure 8a‐2) (Ferrari *et al*., 2012). Following this, the samples were collected and fungal biomass was quantified using the same method as in wheat, but with *protodermal factor 2 (AtPDF2)* as the Arabidopsis reference gene at six dpi with three biological replicates. The data for each study were analysed using a Student *t*‐test.

## Supporting information


**Figure S1** Comparison of promoter DNA sequence variation between NIL‐R, NIL‐S and *Chinese spring TaACT*.
**Figure S2** Comparison of DNA sequence variation between NIL‐R, NIL‐S and *Chinese spring TaACT*. Green underlined indicates 5′ and 3′ regions.
**Figure S3** Purification of bacterial expressed *TaACT*.
**Figure S4** Silencing of the phytoene desaturase (PDS) gene.
**Table S1** List of primers used in the experiments.
**Procedure S1** Detailed procedure followed for development of transgenic Arabidopsis plants over‐expressing *TaACT*.

## References

[pbi12641-bib-0001] Altschul, S.F. , Gish, W. , Miller, W. , Myers, E.W. and Lipman, D.J. (1990) Basic local alignment search tool. J. Mol. Biol. 215, 403–410. 2231712 10.1016/S0022-2836(05)80360-2

[pbi12641-bib-0002] Bai, G. and Shaner, G. (1994) Scab of wheat: prospects for control. Plant Dis. 78, 760–766.

[pbi12641-bib-0003] Bai, G. and Shaner, G. (2004) Management and resistance in wheat and barley to fusarium head blight. Annu. Rev. Phytopathol. 42, 135–161. 15283663 10.1146/annurev.phyto.42.040803.140340

[pbi12641-bib-0004] Bollina, V. , Kumaraswamy, G.K. , Kushalappa, A.C. , Choo, T.M. , Dion, Y. , Rioux, S. , Faubert, D. *et al*. (2010) Mass spectrometry‐based metabolomics application to identify quantitative resistance‐related metabolites in barley against Fusarium head blight. Mole. Plant Pathol. 11, 769–782. 10.1111/j.1364-3703.2010.00643.x PMC6640360 21029322

[pbi12641-bib-0005] Burhenne, K. , Kristensen, B.K. and Rasmussen, S.K. (2003) A new class of N‐Hydroxycinnamoyltransferases purification, cloning, and expression of a barley agmatine coumaroyltransferase (EC 2.3. 1.64). J. Biol. Chem. 278, 13919–13927. 12582168 10.1074/jbc.M213041200

[pbi12641-bib-0056] Cakir, C. and Scofield, S. (2008) Evaluating the ability of the barley stripe mosaic virus‐induced gene silencing system to simultaneously silence two wheat genes. Cereal Research Communications, 36, 217–222.

[pbi12641-bib-0006] Chamarthi, S.K. , Kumar, K. , Gunnaiah, R. , Kushalappa, A.C. , Dion, Y. and Choo, T.M. (2014) Identification of fusarium head blight resistance related metabolites specific to doubled‐haploid lines in barley. Eur. J. Plant Pathol. 138, 67–78.

[pbi12641-bib-0007] Corpet, F. (1988) Multiple sequence alignment with hierarchical clustering. Nucleic Acids Res. 16, 10881–10890. 2849754 10.1093/nar/16.22.10881 PMC338945

[pbi12641-bib-0008] Dexter, J. , Clear, R. and Preston, K. (1996) Fusarium head blight: effect on the milling and baking of some Canadian wheats. Cereal Chem. 73, 695–701.

[pbi12641-bib-0009] Edreva, A. , Velikova, V. and Tsonev, T. (2007) Phenylamides in plants. Russ. J. Plant Physiol. 54, 287–301.

[pbi12641-bib-0010] Facchini, P.J. , Hagel, J. and Zulak, K.G. (2002) Hydroxycinnamic acid amide metabolism: physiology and biochemistry. Can. J. Bot. 80, 577–589.

[pbi12641-bib-0011] Ferrari, S. , Sella, L. , Janni, M. , De Lorenzo, G. , Favaron, F. and D'Ovidio, R. (2012) Transgenic expression of polygalacturonase‐inhibiting proteins in Arabidopsis and wheat increases resistance to the flower pathogen Fusarium graminearum. Plant Biol. 14, 31–38. 21974721 10.1111/j.1438-8677.2011.00449.x

[pbi12641-bib-0012] Fester, T. , Maier, W. and Strack, D. (1999) Accumulation of secondary compounds in barley and wheat roots in response to inoculation with an arbuscular mycorrhizal fungus and co‐inoculation with rhizosphere bacteria. Mycorrhiza, 8, 241–246.

[pbi12641-bib-0013] Gong, X. , Zhang, J. , Hu, J. , Wang, W. , Wu, H. , Zhang, Q. and Liu, J.H. (2015) FcWRKY70, a WRKY protein of Fortunella crassifolia, functions in drought tolerance and modulates putrescine synthesis by regulating arginine decarboxylase gene. Plant, Cell Environ. 38, 2248–2262. 25808564 10.1111/pce.12539

[pbi12641-bib-0014] Gorzolka, K. , Bednarz, H. and Niehaus, K. (2014) Detection and localization of novel hordatine‐like compounds and glycosylated derivates of hordatines by imaging mass spectrometry of barley seeds. Planta, 239, 1321–1335. 24671626 10.1007/s00425-014-2061-y

[pbi12641-bib-0015] Gunnaiah, R. , Kushalappa, A.C. , Duggavathi, R. , Fox, S. and Somers, D.J. (2012) Integrated metabolo‐proteomic approach to decipher the mechanisms by which wheat QTL (Fhb1) contributes to resistance against Fusarium graminearum. PLoS ONE, 7, e40695. 22866179 10.1371/journal.pone.0040695 PMC3398977

[pbi12641-bib-0016] Hamzehzarghani, H. , Paranidharan, V. , Abu‐Nada, Y. , Kushalappa, A. , Mamer, O. and Somers, D. (2008) Metabolic profiling to discriminate wheat near isogenic lines, with quantitative trait loci at chromosome 2DL, varying in resistance to fusarium head blight. Can. J. Plant Sci. 88, 789–797.

[pbi12641-bib-0017] Hoffmann, L. , Besseau, S. , Geoffroy, P. , Ritzenthaler, C. , Meyer, D. , Lapierre, C. , Pollet, B. *et al*. (2004) Silencing of hydroxycinnamoyl‐coenzyme A shikimate/quinate hydroxycinnamoyltransferase affects phenylpropanoid biosynthesis. Plant Cell, 16, 1446–1465. 15161961 10.1105/tpc.020297 PMC490038

[pbi12641-bib-0018] Ishihara, A. , Hashimoto, Y. , Tanaka, C. , Dubouzet, J.G. , Nakao, T. , Matsuda, F. , Nishioka, T. *et al*. (2008) The tryptophan pathway is involved in the defense responses of rice against pathogenic infection via serotonin production. Plant J. 54, 481–495. 18266919 10.1111/j.1365-313X.2008.03441.x

[pbi12641-bib-0019] Janni, M. , Sella, L. , Favaron, F. , Blechl, A. , De Lorenzo, G. and D'Ovidio, R. (2008) The expression of a bean polygalacturonase‐inhibiting proteins in transgenic wheat confers increased resistance to the fungal pathogen Bipolaris sorokiniana. Mol. Plant Microbe Interact. 21, 171–177. 18184061 10.1094/MPMI-21-2-0171

[pbi12641-bib-0020] Kage, U. , Kumar, A. , Dhokane, D. , Karre, S. and Kushalappa, A.C. (2015) Functional molecular markers for crop improvement. Crit. Rev. Biotechnol. 16, 1–14. 10.3109/07388551.2015.1062743 26171816

[pbi12641-bib-0021] Katajamaa, M. , Miettinen, J. and Orešič, M. (2006) MZmine: toolbox for processing and visualization of mass spectrometry based molecular profile data. Bioinformatics, 22, 634–636. 16403790 10.1093/bioinformatics/btk039

[pbi12641-bib-0022] Keller, H. , Hohlfeld, H. , Wray, V. , Hahlbrock, K. , Scheel, D. and Strack, D. (1996) Changes in the accumulation of soluble and cell wall‐bound phenolics in elicitor‐treated cell suspension cultures and fungus‐infected leaves of Solanum tuberosum. Phytochemistry, 42, 389–396.

[pbi12641-bib-0023] Kumar, A. , Karre, S. , Dhokane, D. , Kage, U. , Hukkeri, S. and Kushalappa, A.C. (2015) Real‐time quantitative PCR based method for the quantification of fungal biomass to discriminate quantitative resistance in barley and wheat genotypes to fusarium head blight. J. Cereal Sci. 64, 16–22.

[pbi12641-bib-0024] Kumaraswamy, G.K. , Bollina, V. , Kushalappa, A.C. , Choo, T.M. , Dion, Y. , Rioux, S. , Mamer, O. *et al*. (2011) Metabolomics technology to phenotype resistance in barley against Gibberella zeae. Eur. J. Plant Pathol. 130, 29–43.

[pbi12641-bib-0025] Kushalappa, A.C. and Gunnaiah, R. (2013) Metabolo‐proteomics to discover plant biotic stress resistance genes. Trends Plant Sci. 18, 522–531. 23790252 10.1016/j.tplants.2013.05.002

[pbi12641-bib-0026] Kushalappa, A.C. , Yogendra, K.N. , Sarkar, K. , Kage, U. and Karre, S. (2016a) Gene discovery and genome editing to develop cisgenic crops with improved resistance against pathogen stress. Can. J. Plant Pathol. 38, 1–17. doi:10.1080/07060661.2016.1199597.

[pbi12641-bib-0027] Kushalappa, A.C. , Yogendra, K.N. and Karre, S. (2016b) Plant innate immune response: qualitative and quantitative resistance. Crit. Rev. Plant Sci. 35, 38–55.

[pbi12641-bib-0028] Liu, S. , Pumphrey, M.O. , Gill, B.S. , Trick, H.N. , Zhang, J.X. , Dolezel, J. , Chalhoub, B. *et al*. (2008) Toward positional cloning of Fhb1, a major QTL for Fusarium head blight resistance in wheat. Cereal Res. Commun. 36, 195–201.

[pbi12641-bib-0029] Long, X. , Balcerzak, M. , Gulden, S. , Cao, W. , Fedak, G. , Wei, Y.‐M. , Zheng, Y.‐L. *et al*. (2015) Expression profiling identifies differentially expressed genes associated with the fusarium head blight resistance QTL 2DL from the wheat variety Wuhan‐1. Physiol. Mol. Plant Pathol. 90, 1–11.

[pbi12641-bib-0060] Livak, K.J. and Schmittgen, T.D. (2001) Analysis of Relative Gene Expression Data Using Real‐Time Quantitative PCR and the 2^−ΔΔCT^ Method. methods 25, 402–408. 11846609 10.1006/meth.2001.1262

[pbi12641-bib-0030] Matsuda, F. , Yonekura‐Sakakibara, K. , Niida, R. , Kuromori, T. , Shinozaki, K. and Saito, K. (2009) MS/MS spectral tag‐based annotation of non‐targeted profile of plant secondary metabolites. Plant J. 57, 555–577. 18939963 10.1111/j.1365-313X.2008.03705.x PMC2667644

[pbi12641-bib-0031] McCartney, C. , Somers, D. , Fedak, G. , DePauw, R. , Thomas, J. , Fox, S. , Humphreys, D. *et al*. (2007) The evaluation of FHB resistance QTL introgressed into elite Canadian spring wheat germplasm. Mol. Breed. 20, 209–221.

[pbi12641-bib-0032] McLusky, S.R. , Bennett, M.H. , Beale, M.H. , Lewis, M.J. , Gaskin, P. and Mansfield, J.W. (1999) Cell wall alterations and localized accumulation of feruloyl‐3′‐methoxytyramine in onion epidermis at sites of attempted penetration by Botrytis allii are associated with actin polarisation, peroxidase activity and suppression of flavonoid biosynthesis. Plant J. 17, 523–534.

[pbi12641-bib-0033] Medina‐Puche, L. , Cumplido‐Laso, G. , Amil‐Ruiz, F. , Hoffmann, T. , Ring, L. , Rodríguez‐Franco, A. , Caballero, J.L. *et al*. (2014) MYB10 plays a major role in the regulation of flavonoid/phenylpropanoid metabolism during ripening of Fragaria× ananassa fruits. J. Exp. Bot. 65, 401–417. 24277278 10.1093/jxb/ert377

[pbi12641-bib-0034] Miyagawa, H. , Ishihara, A. , Nishimoto, T. , Ueno, T. and Mayama, S. (1995) Induction of avenanthramides in oat leaves inoculated with crown rust fungus, Puccinia coronata f. sp. avenae. Biosci. Biotechnol. Biochem. 59, 2305–2306.

[pbi12641-bib-0035] Moheb, A. , Ibrahim, R.K. , Roy, R. and Sarhan, F. (2011) Changes in wheat leaf phenolome in response to cold acclimation. Phytochemistry, 72, 2294–2307. 21955620 10.1016/j.phytochem.2011.08.021

[pbi12641-bib-0036] Muroi, A. , Ishihara, A. , Tanaka, C. , Ishizuka, A. , Takabayashi, J. , Miyoshi, H. and Nishioka, T. (2009) Accumulation of hydroxycinnamic acid amides induced by pathogen infection and identification of agmatine coumaroyltransferase in Arabidopsis thaliana. Planta, 230, 517–527. 19521717 10.1007/s00425-009-0960-0

[pbi12641-bib-0037] Muroi, A. , Matsui, K. , Shimoda, T. , Kihara, H. , Ozawa, R. , Ishihara, A. , Nishihara, M. *et al*. (2012) Acquired immunity of transgenic torenia plants overexpressing agmatine coumaroyltransferase to pathogens and herbivore pests. Sci. Rep. 2, 689. 23008754 10.1038/srep00689 PMC3449287

[pbi12641-bib-0038] Ogura, Y. , Ishihara, A. and Iwamura, H. (2001) Induction of hydroxycinnamic acid amides and tryptophan by jasmonic acid, abscisic acid and osmotic stress in barley leaves. Zeitschrift für Naturforschung C, 56, 193–202. 10.1515/znc-2001-3-405 11371008

[pbi12641-bib-0039] Pushpa, D. , Yogendra, K.N. , Gunnaiah, R. , Kushalappa, A.C. and Murphy, A. (2013) Identification of Late Blight Resistance‐Related Metabolites and Genes in Potato through Nontargeted Metabolomics. Plant Mol. Biol. Rep. 32, 1–12.

[pbi12641-bib-0040] von Röpenack, E. , Parr, A. and Schulze‐Lefert, P. (1998) Structural analyses and dynamics of soluble and cell wall‐bound phenolics in a broad spectrum resistance to the powdery mildew fungus in barley. J. Biol. Chem. 273, 9013–9022. 9535889 10.1074/jbc.273.15.9013

[pbi12641-bib-0041] Schmidt, A. , Scheel, D. and Strack, D. (1998) Elicitor‐stimulated biosynthesis of hydroxycinnamoyltyramines in cell suspension cultures of Solanum tuberosum. Planta, 205, 51–55.

[pbi12641-bib-0042] Schweiger, W. , Steiner, B. , Ametz, C. , Siegwart, G. , Wiesenberger, G. , Berthiller, F. , Lemmens, M. *et al*. (2013) Transcriptomic characterization of two major Fusarium resistance quantitative trait loci (QTL), Fhb1 and Qfhs.ifa‐5A, identifies novel candidate genes. Mole. Plant Pathol. 14, 772–785. 10.1111/mpp.12048 PMC3902993 23738863

[pbi12641-bib-0044] Scofield, S.R. and Brandt, A.S. (2012) Virus‐induced gene silencing in hexaploid wheat using barley stripe mosaic virus vectors. Methods in molecular biology (Clifton, N.J.) 894, 93–112. 10.1007/978-1-61779-882-5_7 22678575

[pbi12641-bib-0045] Solovyev, V. , Kosarev, P. , Seledsov, I. and Vorobyev, D. (2006) Automatic annotation of eukaryotic genes, pseudogenes and promoters. Genome Biol. 7, S10. 10.1186/gb-2006-7-s1-s10 PMC1810547 16925832

[pbi12641-bib-0046] Somers, D.J. , Fedak, G. and Savard, M. (2003) Molecular mapping of novel genes controlling Fusarium head blight resistance and deoxynivalenol accumulation in spring wheat. Genome, 46, 555–564. 12897863 10.1139/g03-033

[pbi12641-bib-0047] Steiner, B. , Lemmens, M. , Griesser, M. , Scholz, U. , Schondelmaier, J. and Buerstmayr, H. (2004) Molecular mapping of resistance to Fusarium head blight in the spring wheat cultivar Frontana. Theoret. Appl. Genet. 109, 215–224. 14997302 10.1007/s00122-004-1620-1

[pbi12641-bib-0048] St‐Pierre, B. , Laflamme, P. , Alarco, A.M. and Luca, E. (1998) The terminal O‐acetyltransferase involved in vindoline biosynthesis defines a new class of proteins responsible for coenzyme A‐dependent acyl transfer. Plant J. 14, 703–713. 9681034 10.1046/j.1365-313x.1998.00174.x

[pbi12641-bib-0049] Tohge, T. and Fernie, A.R. (2010) Combining genetic diversity, informatics and metabolomics to facilitate annotation of plant gene function. Nat. Protoc. 5, 1210–1227. 20539294 10.1038/nprot.2010.82

[pbi12641-bib-0050] Wen, W. , Li, D. , Li, X. , Gao, Y. , Li, W. , Li, H. , Liu, J. *et al*. (2014) Metabolome‐based genome‐wide association study of maize kernel leads to novel biochemical insights. Nat. Commun. 5, 3438. 24633423 10.1038/ncomms4438 PMC3959190

[pbi12641-bib-0051] Xiao, J. , Jin, X. , Jia, X. , Wang, H. , Cao, A. , Zhao, W. , Pei, H. *et al*. (2013) Transcriptome‐based discovery of pathways and genes related to resistance against Fusarium head blight in wheat landrace Wangshuibai. BMC Genom. 14, 197. 10.1186/1471-2164-14-197 PMC3616903 23514540

[pbi12641-bib-0052] Ye, J. , Coulouris, G. , Zaretskaya, I. , Cutcutache, I. , Rozen, S. and Madden, T.L. (2012) Primer‐BLAST: a tool to design target‐specific primers for polymerase chain reaction. BMC Bioinform. 13, 134. 10.1186/1471-2105-13-134 PMC3412702 22708584

[pbi12641-bib-0053] Yogendra, K.N. , Pushpa, D. , Mosa, K.A. , Kushalappa, A.C. , Murphy, A. and Mosquera, T. (2014) Quantitative resistance in potato leaves to late blight associated with induced hydroxycinnamic acid amides. Funct. Integr. Genom. 14, 285–298. 10.1007/s10142-013-0358-8 24408130

[pbi12641-bib-0054] Yogendra, K.N. , Kumar, A. , Sarkar, K. , Li, Y. , Pushpa, D. , Mosa, K.A. , Duggavathi, R. *et al*. (2015) Transcription factor StWRKY1 regulates phenylpropanoid metabolites conferring late blight resistance in potato. J. Exp. Bot. 66, 7377–7389. 26417019 10.1093/jxb/erv434 PMC4765800

[pbi12641-bib-0055] Zadoks, J.C. , Chang, T.T. and Konzak, C.F. (1974) A decimal code for the growth stages of cereals. Weed Res. 14, 415–421.

