# Supplementary material for: Identification and characterization of a fusarium head blight resistance gene TaACT in wheat QTL‐2DL
Source: Plant Biotechnol J. 2016 Nov 4;15(4):447–57. doi: 10.1111/pbi.12641 (PMC5362683; doi:10.1111/pbi.12641)
Supplement: Supplementary file 1 — Figure S1 Comparison of promoter DNA sequence variation between NIL‐R, NIL‐S and Chinese spring TaACT. Figure S2 Comparison of DNA sequence variation between NIL‐R, NIL‐S and Chinese spring TaACT. Green underlined indicates 5′ and 3′ regions. Figure S3 Purification of bacterial expressed TaACT. Figure S4 Silencing of the phytoene desaturase (PDS) gene. Table S1 List of primers used in the experiments. Procedure S1 Detailed procedure followed for development of transgenic Arabidopsis plants over‐expressing TaACT. [file PBI-15-447-s001.docx]

**Identification and characterization of a fusarium head blight resistance gene *TaACT* in wheat QTL-2DL**

Udaykumar Kage, Shailesh Karre, Ajjamada C. Kushalappa, and Curt McCartney


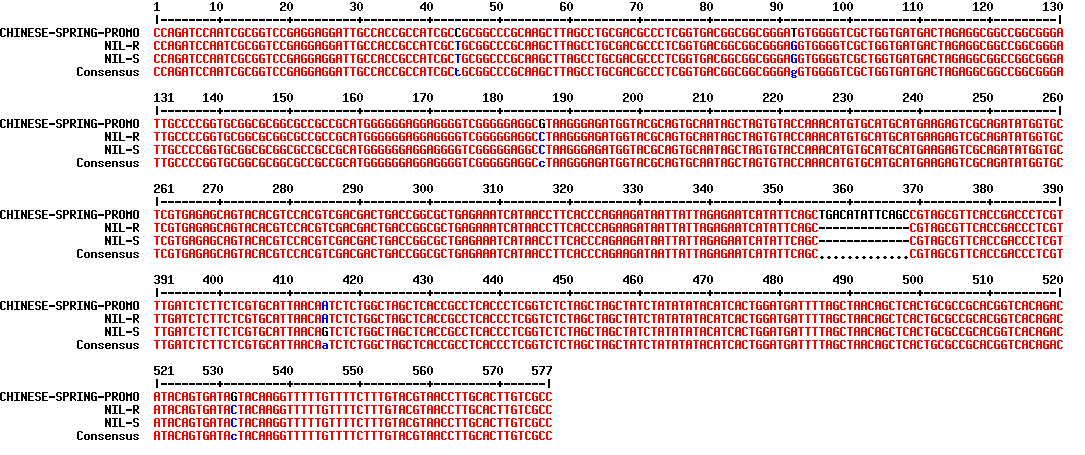


Figure S1: Comparison of promoter DNA sequence variation between NIL-R, NIL-S and *Chinese spring* *TaACT.*


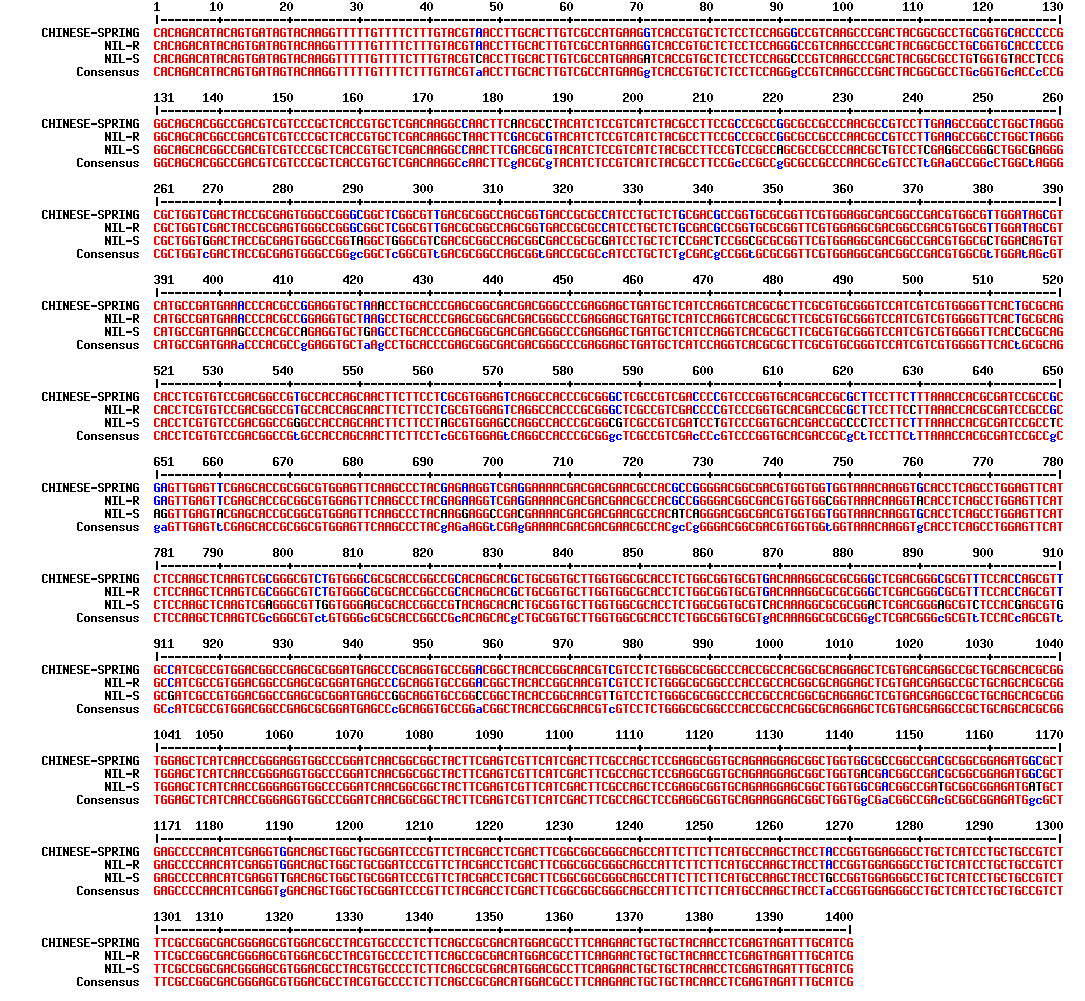


Figure S2: Comparison of DNA sequence variation between NIL-R, NIL-S and *Chinese spring* *TaACT.* Green underlined indicates 5´ and 3´ regions.


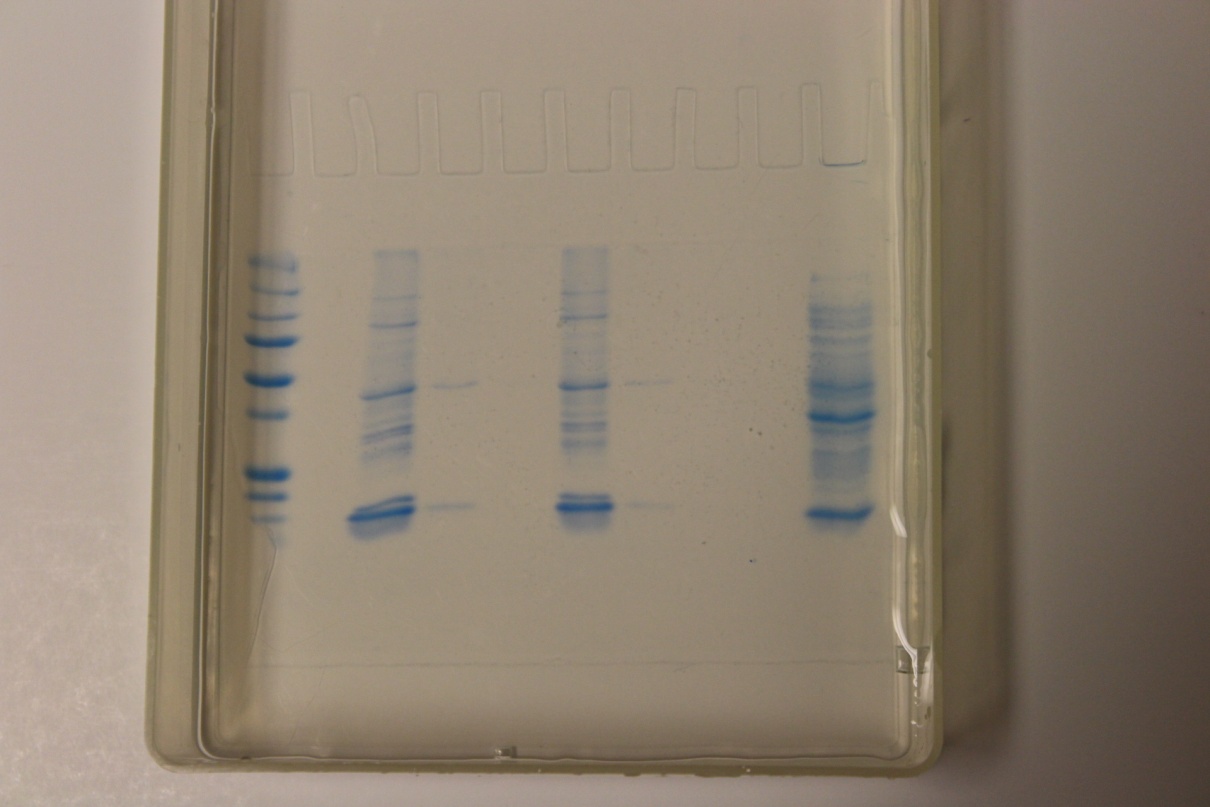


L S1 S2 S3 S4 S5

50kDa

Figure S3:Purification of bacterial expressed *TaACT*. L is Protein marker, S1, S2 & S3, S4 are sequential eluted fractions of recombinant *2DL-TaACT*, S5 is Crude *TaACT* extract. This is experimental evidence to prove the predicted TaACT protein size (~48kDa) and to show which is comparable to the earlier reported barley HvACT protein size (~48kDa) (Burhene et al., 2003).


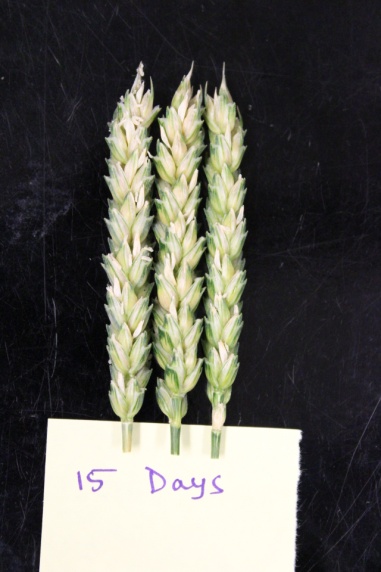


Figure S4: Silencing of the phytoene desaturase (PDS) gene. Photograph indicates the phenotypes of resistant wheat plants infected with BSMV*_PDS_*

Table S1: List of primers used in the experiments

|  | **Gene/Primer Name** | **Forward primer (5´ - 3´)** | **Reverse primer (5´ - 3´)** |
| --- | --- | --- | --- |
| **Fungal biomass** | *Tri6* | TCTTTGTGAGCGGACGGGACTTTA | TGTTGGTTTGTGCTTGGACTCAT |
| **Gene Sequencing** | *TaACT* | CACAGACATACAGTGATAGTA CAAGG | CGATGCAAATCTACTCGAGG |
| **Promoter sequencing** | *TaACT promoter* | CCAGATCCAATCGCGGTCCGAGGA | GGCGACAAGTGCAAGGTTA |
| **RT-qPCR** | *TaACTq* | ACCACGCGATCCGCCGCGAG | CGGCGTGGCGTTCGTCGTCGTT |
| **Protein expression** | *TaACT protein* | GACAGATCTATGAAGGTCACCGTGCTCTC | GGTGAATTCCTACTCGAGGTTGTAGCAGC |
| **VIGS fragment** | *TaACT VIGS* | GAGGTGGACAGCTGGCTG | GGCACTGCAGATACATTTCAAAAC |
| **VIGS Expression** | *TaACT VIGS EXP* | ACCACGCGATCCGCCGCGAG | CGGCGTGGCGTTCGTCGTCGTT |
| **Over expression** | *TaACT cDNA* | ATGAAGGTCACCGTGCTCTC | CTACTCGAGGTTGTAGCAG |
| **Reference genes** | *TaActin* | ACCTTCAGTTGCCCAGCAAT | CAGAGTCGAGCACAATACCAGTTG |
|  | *AtPDF2* | TCATTCCGATAGTCGACCAAG | TTGATTTGCGAAATACCGAAC |
| **SSR Markers** | *wmc245* |  |  |
|  | *gwm539* | CTGCTCTAAGATTCATGCAACC | GAGGCTTGTGCCCTCTGTAG |
|  | *gwm608* | ACATTGTGTGTGCGGCC | GATCCCTCTCCGCTAGAAGC |

**Procedure S1: Detailed procedure followed for development of transgenic Arabidopsis plants over-expressing *TaACT***

For *TaACT* functional complementation study, the coding sequence was amplified from cDNA and cloned into of ZeBaTA based expression vector (Chen et al., 2009). Constructs were introduced into Agrobacterium tumefaciens strain GV3101 by freeze thaw method (Weigel and Glazebrook, 2005). Agrobacterium (GV3101) carrying the recombinant constructs was cultured in 5ml liquid LB added with 50 mg L^−1^ hygromycin, 1 ml of this feeder culture was used to inoculate 500 ml of liquid LB containing 50 mg L^−1^ hygromycin and grown at 28°C for 16 - 24 hr till it reaches OD = 1.5. The Agrobacterium was separated from the LB medium by centrifugation at 4000rpm for 10 min at room temperature, and gently cell pellets were re-suspended in 5 % sucrose solution, to this mixture 0.02 % Tween-20 was added and mixed well. Arabidopsis *AtACT* gene T-DNA insertion mutant plants (At5g61160) deficient in coumaroylagmatine and coumaroylputrescien, were grown and at flowering stage, plants were transformed by floral dip method. Transformed plants were covered with plastic cover for 24 hr to maintain high humidity. After maturity, seeds were collected and used for screening of primary transformants on selection plates (Half a strength of Murashige and Skoog medium + 50 mg L^−1^ hygromycin + 100 mg L^−1^Carbenicillin) after sterilizing them with 70 % ethanol and 50 % bleach. Screening of transgenic plants and confirmation was according to previous reports (Zhang et la., 2006 and Bent, 2006).

**References**

Bent, Andrew. "Arabidopsis thaliana floral dip transformation method."*Agrobacterium Protocols*. Humana Press, 2006. 87-104.

Burhenne, K., Kristensen, B.K. and Rasmussen, S.K. (2003) A New Class of N-Hydroxycinnamoyltransferases purification, cloning, and expression of a barley agmatine coumaroyltransferase (EC 2.3. 1.64). *Journal of Biological Chemistry***278**, 13919-13927.

Chen, S., Songkumarn, P., Liu, J., & Wang, G.L. (2009). A versatile zero background T-vector system for gene cloning and functional genomics. *Plant physiology, 150*(3), 1111-1121.

Weigel, D., & Glazebrook, J. (2005). Transformation of agrobacterium using the freeze-thaw method. *CSH protocols, 2006*(7), 1031-1036.

Zhang, X., Henriques, R., Lin, S.-S., Niu, Q.-W., & Chua, N.-H. (2006). Agrobacterium-mediated transformation of Arabidopsis thaliana using the floral dip method. *Nature protocols, 1*(2), 641-646.
